# Processing of Kansui Roots Stir-Baked with Vinegar Reduces Kansui-Induced Hepatocyte Cytotoxicity by Decreasing the Contents of Toxic Terpenoids and Regulating the Cell Apoptosis Pathway

**DOI:** 10.3390/molecules19067237

**Published:** 2014-06-03

**Authors:** Xiaojing Yan, Li Zhang, Jianming Guo, Yudan Cao, Erxin Shang, Yuping Tang, Anwei Ding, Jin-Ao Duan

**Affiliations:** Jiangsu Key Laboratory for High Technology Research of TCM Formulae, Nanjing University of Chinese Medicine, Nanjing 210023, China; E-Mails: yanxiaojing963@163.com (X.Y.); njuguo@njutcm.edu.cn (J.G.); raindc@163.com (Y.C.); shex@njutcm.edu.cn (E.S.); awding105@163.com (A.D.); dja@njutcm.edu.cn (J.D.)

**Keywords:** kansui, kansui stir-baked with vinegar, hepatotoxicity, intrinsic pathway apoptosis, high content screening, differentiating components

## Abstract

*Euphorbia kansui* is a Traditional Chinese Medicine widely used for the treatment of oedema, ascites and asthma. However, its serious hepatotoxicity hinders its safe clinical application. The process of stir-baking with vinegar is regularly used to reduce the toxicity of kansui. Up till now, the exact mechanism of the reduction in hepatotoxicity of kansui stir-baked with vinegar has been poorly defined. In this study, decreased contents of five diterpene and one triterpene in kansui (GS-1) after stir-baking with vinegar (GS-2) was investigated by UPLC-QTOF/MS. Flow cytometry and Hoechst staining were used to show that the stir-baking with vinegar process reduces kansui-induced cell apoptosis. Furthermore, the result also indicated that kansui stir-baked with vinegar protects LO2 cells from apoptosis by increasing the cell mitochondrial membrane potential (*ΔΨ*m), decreasing the release of cytochrome c and inhibiting the activities of caspase-9 and caspase-3 as evidenced by means of high content screening (HCS), ELISA and western blotting. These results suggested that the stir-baking vinegar could reduce the hepatotoxicity of kansui by effectively decreasing the contents of toxic terpenoids and inhibiting the intrinsic pathway of hepatocyte cell apoptosis. In conclusion, the study provided significant data for promoting safer and better clinical use of this herb.

## 1. Introduction

The safety of Traditional Chinese Medicine (TCMs) has drawn increasing attention in China. The roots of *Euphorbia* *kansui* T. N. Liou ex T. P. Wang (called kansui), recorded in *Shennong-Bencao*, has been used in China for centuries for multiple medical applications, in particular for oedema, ascite, and asthma [1]. Many studies showed that kansui also exhibits promising effects in the treatment of cancer [1,2,3,4,5], pancreatitis [6,7,8] and intestinal obstruction [9,10]. However, the clinical application of kansui has some practical disadvantages due to inflammation, skin, oral, and gastrointestinal irritation and promotion of tumors [11,12,13,14,15]. Recent studies have also shown that kansui could lead to hepatic injury [15,16,17,18,19,20]. The stir-baking with vinegar process is regularly used in Traditional Chinese Medicine to reduce the toxicity of kansui, and our previous studies have also shown that stir-baking with vinegar reduced the toxicity of kansui *in vitro* and *in vivo* [13,15,21]. However, the mechanism of the reduction in hepatoxicity of kansui stir-baked with vinegar is still poorly defined.

The human normal hepatocyte cell line LO2, also known as “L-O2” or “HL-7702”, derives from primary normal human hepatocytes and has an epithelioid cell phenotype. The LO2 cell line was generated in 1980 by Dr Xiuzhen Ye and her colleagues from the Chinese Academy of Sciences (Shanghai, China). In their study, the ten early generations of LO2 cells were found to maintain the biological features and ultrastructures of normal adult hepatocytes [22,23]. In the following studies, this cell line has been widely used as normal human liver cell line [24,25,26,27,28,29,30,31,32]. Thus, the early ten generations of LO2 cells were chosen in our study to investigate the effects of kansui and kansui stir-baked with vinegar on LO2 cell cycle and apoptosis. Our previous studies have demonstrated that stir-baking with vinegar could reduce the hepatotoxicity of kansui by affecting LO2 cell cycle and apoptosis [19]. Therefore, the study attempted to investigate the molecular mechanisms relating to the reduction in LO2 cells apoptosis of kansui stir-baked with vinegar.

Several terpenoids and phenolic derivatives have been isolated from kansui and identified in previous phytochemical investigations [12,33,34,35,36,37,38]. On the one hand, the terpenoids in kansui, including jatrophane-type diterpenoids, ingenane-type diterpenoids and triterpenoids, exhibited comprehensive bioactivities such as anti-tumor [1,2,4,5,35], anti-allergy [39], anti-virus [12,40], and anti-nematodal activity [41]. On the other hand, some of the terpenoids, including jatrophane-type diterpenoids and 8-ene-7-one triterpenoids exhibited serious hepatotoxicity in LO2 cells [20].

In Ogasawara research group established from biochemical, histological and electron microscope analyses that severe liver damage might result from apoptosis, and apoptosis plays a critical role in the pathological processes of various liver diseases [42,43]. Apoptosis can be initiated by two alternative signaling pathways: the death receptor-mediated extrinsic apoptotic pathway and the mitochondrion-mediated intrinsic apoptotic pathway [44,45]. The intrinsic pathway involves the cell oxidative stress that triggers the mitochondria-dependent pathway, resulting in the induction of decreased mitochondrial membrane potential (*ΔΨ*m), cytochrome c release from mitochondria into cytosol and the activation of caspase cascade [45].

The current study investigated the variation of the chemical compounds in kansui before and after stir-baking with vinegar and the effect on cell apoptosis to explore the mechanism of reduction of hepatotoxicity of kansui stir-baked with vinegar. The findings show that kansui stir-baked with vinegar could reduce the hepatotoxicity of kansui by effectively decreasing the content of toxic terpenoids and inhibiting the intrinsic pathway of hepatocyte cell apoptosis via blockade of mitochondrial cytochrome c release and caspase activation and this research is to lay the foundation for further demonstrating detoxication mechanism of kansui stir-baked with vinegar.

## 2. Results and Discussion

### 2.1. UPLC-QTOF/MS Analysis and Identification of the Most Differentiating Components between Kansui (GS-1) and Kansui Stir-Baked with Vinegar (GS-2)

**Figure 1:**
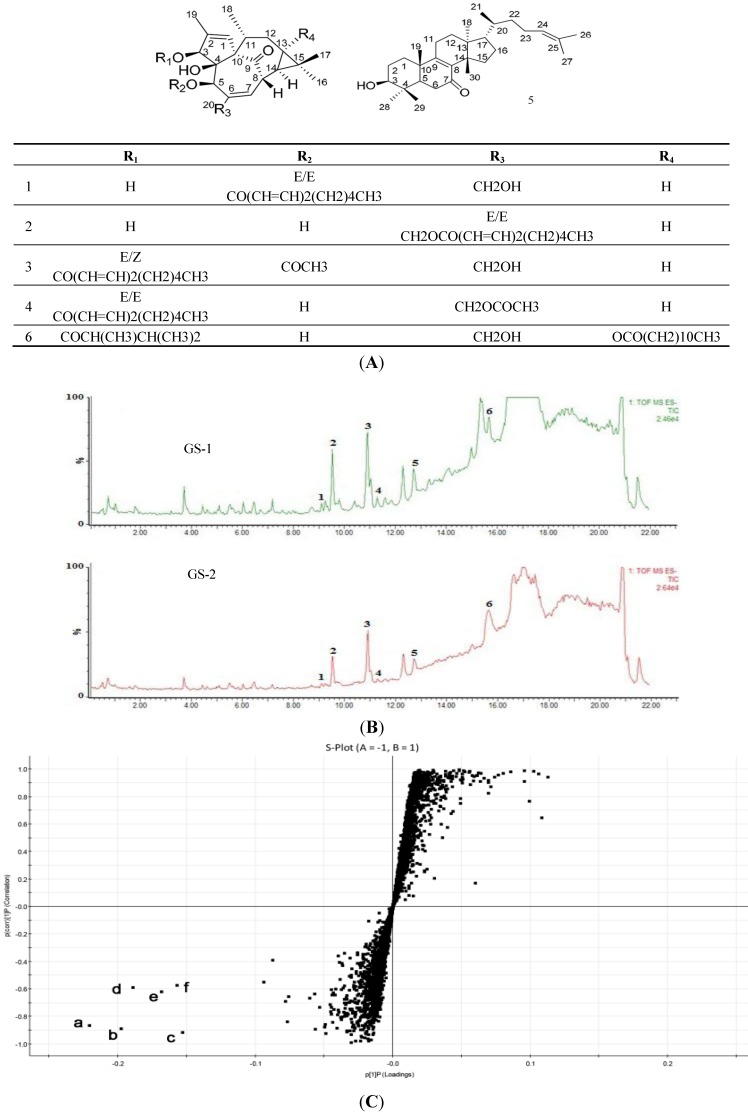
The five diterpene and one triterpene as the most differentiating components were analysized by UPLC-QTOF/MS. (**A**) The chemical structures of the six reference compounds. (**B**) UPLC-negative-ion ESI-MS total ion-current (TIC) profile of GS-1 and GS-2 (peaks **1**–**6** are the peaks of the six reference compounds). (**C**) OPLS-DA/S-plot of GS-1 and GS-2 using Pareto scaling with mean centering. **a**: (RT 15.69 min, *m/z* 643.4228); **b**: (RT 9.11 min, *m/z* 497.2883); **c**: (RT 11.29 min, *m/z* 539.3015); **d**: (RT 10.91 min, *m/z* 539.3026); **e**: (RT 9.53 min, *m/z* 497.2889); **f**: (RT 12.71 min, *m/z* 439.3562).

The base peak intensity (BPI) chromatograms of GS-1 and GS-2 are shown in Figure 1B. To globally evaluate the chemical variation between GS-1 and GS-2, these two samples were subjected to UPLC-QTOF/MS analysis, and the datasets of retention time (RT)-*m/z* pairs, ion intensities and sample codes were further processed with Markerlynx XS. The datasets was subjected to extended statistics using EZinfo. In order to find out the components contributing most to the differences, the extended statistical analysis was used to provide an S-plot (Figure 1C), in which each point represents an ion RT–*m/z* pair; the X axis represents variable contribution, and the further the ion RT–*m/z* pair point departs from zero, the more the ion contributes to the difference between two groups; the Y axis represents variable confidence, and the further the ion RT–*m/z* pair points departs from zero, the higher the confidence level of the ion is for the difference between two groups. Therefore, the RT–*m/z* pair points at the two ends of “S” represent those components contributing most to the difference between these two kinds of samples with most confidence, which could be regarded as the most differentiating components between GS-1 and GS-2.

According to the S-plot (Figure 1C), the six ions **1**–**6** at the bottom left corner of “S” are the ions in samples of GS-1 which contributed most to the difference between GS-1 and GS-2. It was found that the intensities of ions **a** (RT 15.69 min, *m/z*643.4228), **b** (RT 9.11 min, *m/z* 497.2883), **c** (RT 11.29 min, *m/z* 539.3015), **d** (RT 10.91 min, *m/z* 539.3026), **e** (RT 9.53 min, *m/z*497.2889), and **f** (RT 12.71 min, *m/z*439.3562) were higher in GS-1 than GS-2, which indicated that the six components correlated to ions **a**-**f** could be used as potential characteristic markers to distinguish GS-1 and GS-2. They were identified by the high resolution ESI-Q-TOFMS and comparison with the reference compounds (Figure 1A). The results are summarized in Table 1, which shows that the mass accuracy for all assigned components was less than 5 ppm compared with the empirical molecular formulas with those of the published compounds from kansui. Furthermore, the identities of 5-*O*-(2'*E*,4'*E*-decadienoyl)ingenol (**1**), 20-*O*-(2'*E*,4'*E*-decadienoyl)ingenol (**2**), 3-*O*-(2'*E*,4'*Z*-decadienoyl)-5-*O*-acetylingenol (**3**), 3-*O*-(2'*E*,4'*E*-decadienoyl)-20-*O*-acetylingenol (**4**), *epi*-kansenone (**5**), and 3-*O*-(2,3-dimethylbutanoyl)-13-*O*-dodecanoylingenol (**6**) were further confirmed by comparing the mass/UV spectra and retention times with those of the reference compounds. As regards the identification of the most differentiating components between GS-1 and GS-2, some prominent ions were found to correspond to deprotonated molecular ions for all of the components. The ions **a-f** correlated to compounds **6**, **1**, **4**, **3**, **2** and **5**, respectively. These differentiating components between GS-1 and GS-2 are well-known toxic constituents in kansui [20], a drop-off of their contents in GS-2 might well interpret that stir-baking with vinegar could reduce the toxicity of kansui.

**Table 1:** The identification and comparison of the most differentiating components between GS-1 and GS-2.

Peak No.	RT (min)	HR-mass (*m/z*)	Tolerance (ppm)	Molecular Formula	Assigned Identity	A		Rate of Change (%)
						GS-1	GS-2	
**1**	9.11	497.2883 (**b**)	−4.0	C_30_H_42_O_6_	5-*O*-(2'*E*,4'*E*-decadienoyl)ingenol	300	236	24.67
**2**	9.53	497.2889 (**e**)	−2.8	C_30_H_42_O_6_	20-*O*-(2'*E*,4'*E*-decadienoyl)ingenol	245	195	20.41
**3**	10.91	539.3026 (**d**)	3.2	C_32_H_44_O_7_	3-*O*-(2'*E*,4'*Z*-decadienoyl)-5-*O*-acetylingenol	414	355	14.25
**4**	11.29	539.3015 (**c**)	1.1	C_32_H_44_O_7_	3-*O*-(2'*E*,4'*E*-decadienoyl)-20-*O*-acetylingenol	330	278	15.75
**5**	12.71	439.3562 (**f**)	−3.2	C_30_H_48_O_2_	*epi*-kansenone	802	683	14.84
**6**	15.69	643.4228 (**a**)	2.8	C_38_H_60_O_8_	3-*O*-(2,3-dimethylbutanoyl)-13-*O*-dodecanoylingenol	942	358	62.00

**a**–**f**: the ions in samples which contributed most to the difference between GS-1 and GS-2.

### 2.2. Effects on the Rate of Cell Apoptosis

**Figure 2:**
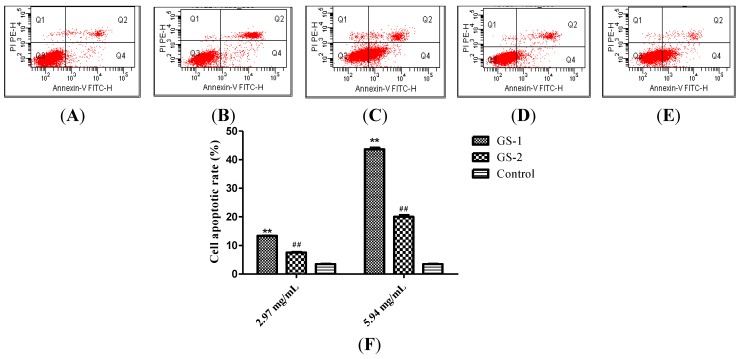
Apoptosis assay using flow cytometer after Annexin V-FITC/PI staining. Control (**A**); LO2 cells were treated with GS-1 at the concentration of 2.97 (**B**) and 5.94 mg/mL (**C**); LO2 cells were treated with GS-2 at the concentration of 2.97 (**D**) and 5.94 mg/mL (**E**); (**F**) Quantification of apoptotic cells, LO2 cells were treated with GS-1 and GS-2 at the concentration of 2.97 mg/mL and 5.94 mg/mL. Values expressed as mean ± SD from three independent experiments, * *p* < 0.05; and ** *p* < 0.01 *vs.* Control; ^#^ *p* < 0.05; and ^##^ *p* < 0.01 *vs.* GS-1.

Cells in the control, GS-1 and GS-2 groups were subjected to Annexin-V staining and PI staining and analyzed by flow cytometer. As shown in Figure 2, compared with control group, the apoptotic cells significantly increased (*p* < 0.01) at the concentration of 2.97, 5.94 mg/mL in a dose-dependent manner in the GS-1 group. Compared with the GS-1 group, the apoptotic cells significantly decreased (*p* < 0.01) in a dose-dependent manner in GS-2 group. Thus, the results demonstrated that kansui could induce LO2 cell apoptosis, and the stir-baking the roots with vinegar could significantly reduce the LO2 cell apoptosis, which indicated that the hepatic toxicity induced by kansui is decreased after stir-baking with vinegar by attenuating the LO2 cell apoptosis.

### 2.3. Effects on Nuclear Fragmentation and the Accumulation of Mitochondrial Mass

**Figure 3:**
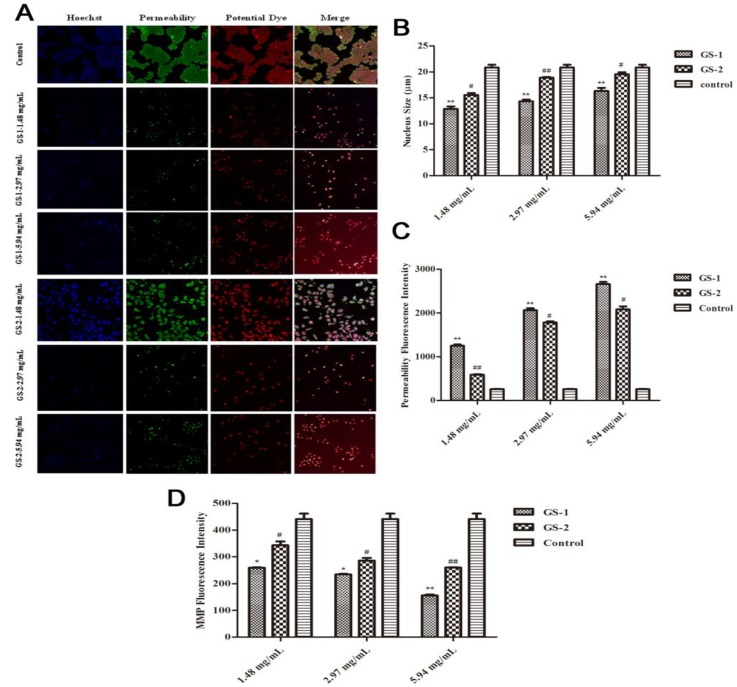
Effects of LO2 cells treated with GS-1 and GS-2 on mitochondrial pathway of apoptosis at the concentration of 1.48, 2.97, 5.94 mg/mL for 48 h and then fixed and stained with the Mutiparameter Cytotoxicity 3 kit, respectively. (**A**) Images of cells were taken by the high content screening (HCS) Assay Scan VTI Reader (×100). (**B**) The average cell nuclear size was quantified and analyzed by the HCS Assay Scan software. (**C**) The cell membrane permeability was reflected by the average intensity of fluorescent dyes permeabilized through cell membranes. (**D**) The cell mitochondrial membrane potential was reflected by the average intensity of mitochondrial membrane potential fluorescent dyes. Values expressed as mean ± SD from three independent experiments, * *p* < 0.05; and ** *p* < 0.01 *vs.* Control; ^#^ *p* < 0.05; and ^##^ *p* < 0.01 *vs.* GS-1.

To further study cell apoptosis, cellular morphological changes was evaluated and the specific mechanism by which the kansui stir-baked with vinegar reduces kansui-induced hepatocyte apoptosis was explored using an emerging methodology known as high content screening. Nuclear, membrane permeability and mitochondrial membrane potential (*ΔΨ*m) were co-stained with fluorescent dyes, and then images were taken with an HCS assay scan VTI Reader. Data derived from these images were analyzed by the HCS software. The results (Figure 3) suggested that mitochondrial pathway is involved in the kansui stir-baked with vinegar to reduce kansui-induced hepatocyte apoptosis. During this process, some nuclei fragmented into multiple subnuclei, and membrane permeability and changes in mitochondrial membrane potential (*ΔΨ*m) were observed (Figure 3A). Compared with cells in control group, after cells were exposed to GS-1, the average cell nucleus size was smaller (*p* < 0.01), the average cell membrane permeability fluorescent intensity was significantly increased (*p* < 0.01) and the average cell mitochondrial membrane potential (*ΔΨ*m) fluorescent intensity was significantly decreased (*p* < 0.05) in a dose-dependent manner. Compared with GS-1 group, in the GS-2 group sample, however, the cells were effectively protected from nuclear fragmentation (*p* < 0.05), and the average cell membrane permeability fluorescent intensity was significantly decreased (*p* < 0.05), while the average cell mitochondrial membrane potential (*ΔΨ*m) fluorescent intensity was significantly increased (*p* < 0.05) in a dose-dependent manner (Figure 3B,C).

### 2.4. Effects on Cytochrome c Release from Mitochondria into the Cytoplasm

**Figure 4:**
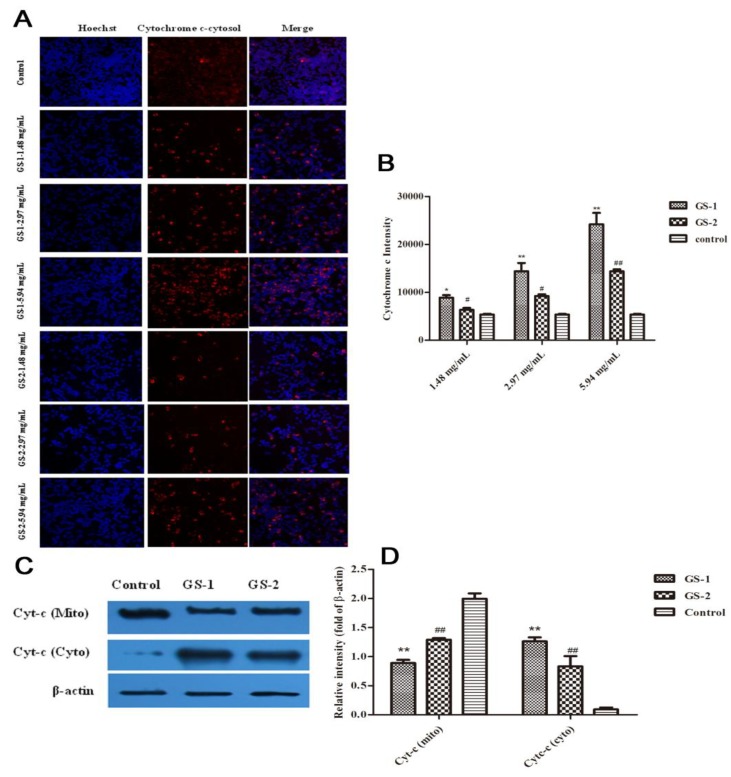
Effects of LO2 cells treated with GS-1 and GS-2 on the release of cytochrome c from mitochondria at the concentration of 2.97, 5.94 mg/mL for 48 h, respectively. (**A**) Images stained with cytochrome c fluorescent dye were taken by the high content screening (HCS) assay scan VTI Reader (×100). (**B**) The release of cytochrome c from mitochondria was reflected by the average cytochrome c intensity of fluorescent dye. (**C**,**D**) The Effects of GS-1 and GS-2 on expression of Cytc (mito) and Cytc (cyto) protein in LO2 cells detected by western blotting. Values expressed as mean ± SD from three independent experiments, * *p* < 0.05; and ** *p* < 0.01 *vs.* Control; ^#^ *p* < 0.05; and ^##^ *p* < 0.01 *vs.* GS-1.

Cytochrome c plays a key role in regulating apoptosis [46,47]. And its release is widely used as a measurement in targeting apoptosis. To better understand the molecular mechanisms in mitochondrial signaling pathway, whether kansui stimulates the release of cytochrome c into the cytosolic fraction of LO2 cells was determined. Images of cytosolic cytochrome c from mitochondria were detected by high content screening assay (Figure 4A). Compared with cells in the control group, in GS-1 group, the average cytochrome c release fluorescent intensity was significantly increased (*p* < 0.05) in a dose-dependent manner. Compared with GS-1 group, after cells were exposed to GS-2, the average cytochrome c release fluorescent intensity was significantly decreased (*p* < 0.05) in a dose-dependent manner (Figure 4B). Subsequently, western blotting was conducted to detect the cytochrome c in both the mitochondrial and cytosolic fractions (Figure 4C). Based on our studies, the level of cytochrome c significantly decreased (*p* < 0.01) in the mitochondrial fraction and significantly increased (*p* < 0.01) in cytosolic fraction after treatment with GS-1 in LO2 cells compared with control group were observed. Conversely, the results also demonstrated that compared with GS-1 group, in GS-2 groups, the cytochrome c level significantly increased in mitochondrial fraction and decreased in the cytosolic fraction (Figure 4D).

### 2.5. Effects on Caspase-9 and Caspase-3 Activation

**Figure 5:**
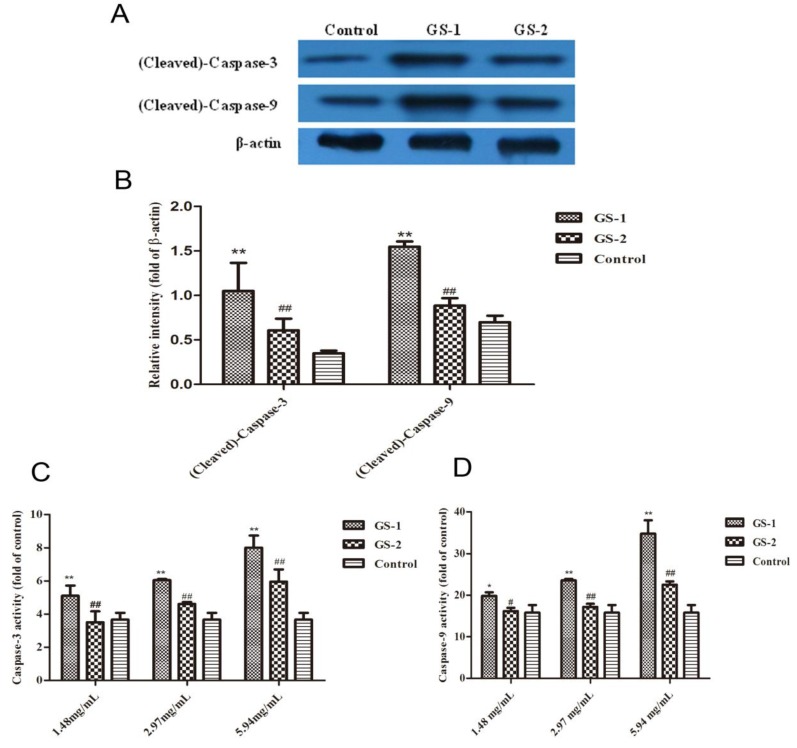
The expression of cleaved-caspase-3 and cleaved-caspase-9 protein in LO2 cells detected by western blotting (**A**,**B**) and the activation of caspase-3 (**C**) and caspase-9 (**D**) were assayed by Elisa according to the manufacturer’s instructions. Values expressed as mean ± SD from three independent experiments, * *p* < 0.05; and ** *p* < 0.01 *vs.* Control; ^#^ *p* < 0.05; and ^##^ *p* < 0.01 *vs.* GS-1.

To gain further insight into the intrinsic pathway of cell apoptosis, the expression of cleaved-caspase-3 and cleaved-caspase-9 protein were measured using western blotting analysis. Results showed that the levels of cleaved-caspase-3 and cleaved-caspase-9 significantly increased in GS-1 group compared with the control group (*p* < 0.01). By contrast, compared with GS-1 group, levels of cleaved-caspase-3 and cleaved-caspase-9 significantly decreased after treatment of GS-2 (*p* < 0.01) (Figure 5A,B). Next, the activation of caspase-3 and caspase-9 were detected by Elisa assay (Figure 5C,D). Compared with cells in control group, after cells were treated with GS-1, the activities of caspase-3 and caspase-9 were significantly increased in a dose-dependent manner (*p* < 0.05). However, compared with GS-1 group, after cells were treated with GS-2, the activities of caspase-3 and caspase-9 were significantly decreased in a dose-dependent manner (*p* < 0.05).

## 3. Experimental

### 3.1. Plant Material

The roots of *Euphorbia kansui* T. N. Liou ex T. P. Wang were collected in October 2010 from Shanxi Province, China, and identified by Professor Chungen Wang (Department of Pharmacognosy, School of Pharmacy, Nanjing University of Chinese Medicine, Nanjing, China). A voucher specimen (No.NJUTCM-20100920) was deposited in the Herbarium of Nanjing University of Chinese Medicine, Nanjing, China.

### 3.2. Preparation of Sample Solutions

The unprocessed kansui (GS-1): 100 g of cleaned kansui (with the same size) was crushed into powder (24-mesh, yield: 99.3%); the kansui stir-baked with vinegar (GS-2): 100 g of cleaned kansui (with the same size) was put into beaker, mixed with 30 g vinegar, placed in a wok at 260 °C, and stir-baked until the vinegar was fully exhausted and slight scorched spots appeared. The sample was crushed into powder after cooling down (24-mesh, the yield: 96.6%).

Samples (25 g) of GS-1 and GS-2 were weighed, and then extracted with 95% EtOH (500 mL) in a 50 °C water bath for 3 h respectively, and this process was repeated eight times. The supernatants were combined and the solvent were removed under reduced pressure. The residue was partitioned with ethyl acetate eight times to provide the EtOAc fraction. The EtOAc fraction was dissolved in MeOH (100 mL), and 5 mL of the solution was used for UPLC assay (passed through a 0.22 μm filter membrane before injection), and the other 95 mL was concentrated under reduced pressure, then the residue dissolved into DMSO as a 4.75 g (crude drug)/mL stock solution, sterilized through a 0.22 μm filter membrane and subsequently diluted into 1.48, 2.97, 5.94, 11.88 and 23.75 mg (crude drug)/mL of serial solutions, respectively.

### 3.3. UPLC-QTOF/MS Experiments

UPLC was performed using a Waters ACQUITY™ UPLC™ system (Waters Corp., Milford, MA, USA), equipped with a binary solvent delivery system, and an auto-sampler. Chromatographic separation was performed on a Thermo C_18_ column (2.1 mm × 100 mm, 1.7 μm). The mobile phase was composed of A (water and 0.1% formic acid) and B (acetonitrile) with a gradient elution: 40%–80% B for 0–8 min, 80%–99% B for 8–17 min, 99% B for 17–20 min, 99%–40% B for 20–21 min, and 40% B for 21–22 min. The flow rate of the mobile phase was 0.4 mL/min, and the column temperature was maintained at 35 °C. Two cycles of weak and strong solvent washing of the injecting system were carried out in-between injections. The injection volume was 5 μL, and the column eluents directly flowed into the mass spectrometer. The mass spectrometry was performed on a Waters Q-TOF mass spectrometer (Micromass, Milford, MA, USA). The settings in negative-ion modes were: capillary potential 3.0 kV, cone potential 30.0 V, source temperature 120 °C, desolvation temperature 350 °C, cone gas flow 50 L/h, desolvation gas flow 600 L/h. The full-scan mass spectra were recorded in the range *m*/*z* 100 to 1,000 Da. All the analyses were operated by using MassLynx 4.1 Software (Waters).

In our study, 5-*O*-(2'*E*,4'*E*-decadienoyl)ingenol (**1**), 20-*O*-(2'*E*,4'*E*-decadienoyl)ingenol (**2**), 3-*O*-(2'*E*,4'*Z*-decadienoyl)-5-*O*-acetylingenol (**3**), 3-*O*-(2'*E*,4'*E*-decadienoyl)-20-*O*-acetylingenol (**4**), *epi*-kansenone (**5**), and 3-*O*-(2,3-dimethylbutanoyl)-13-*O*-dodecanoylingenol (**6**) were chosen as reference compounds (Figure 1); they were isolated from the roots of *E. kansui* in our laboratory and were identified on the basis of spectral analysis [20]. Under the UPLC conditions described above, their retention times (*t*_R_) were 9.11 (**1**), 9.53 (**2**), 10.91 (**3**), 11.29 (**4**), 12.71 (**5**) and 15.69 (**6**), respectively.

### 3.4. Multivariate Data Analysis for the UPLC-QTOF/MS Data

The UPLC-QTOF/MS data of all determined samples were analyzed by MarkerLynx XS software (Waters, Manchester, UK) to identify the potential discriminated variables. For data collection, the method parameters were set as follows: retention time (RT) in the range of 0–22 min and mass in the range of 100–1,000 Da, with a mass tolerance of 0.05 Da. ApexTrack™ peak integration was used to detect chromatographic peaks. The parameters of width at 5% height and peak-to-peak baseline noise were automatically calculated. For the collection parameters, intensity threshold was set at 10 counts, mass window 0.05 Da, RT window 0.1 min. No specific mass or adduct was excluded. Isotopic peaks were excluded for analysis. Moreover, a list of the intensities of the peaks detected was generated by using RT and mass data (*m*/*z*) pairs as the identifier of each peak. An arbitrary ID was assigned to each of these RT-*m*/*z* pairs in the order of their UPLC elution for data alignment. The process was repeated for each run. After completion, the correct peak intensity data for each RT-*m/z* pair of the entire batch of samples were aligned in the final data table. The ions that showed the same RT (with a tolerance of 0.1 min) and *m/z* value (with a tolerance of 0.05 Da) in different samples were considered as the same ion. For those peaks hard to detect in the sample, the ion intensities were documented as zero in the final data table. Before submitted for multivariate analyses, the ion intensities for each detected peak were normalized against the sum of the peak intensities within that sample using MarkerLynx software. Afterwards, the resulting three-dimensional data comprising of peak number (RT-*m/z* pair), sample name and ion intensity were analyzed by orthogonal partial least squared discriminant analysis (OPLS-DA) within the EZInfo software.

### 3.5. Cell Line and Cell Culture

Human normal liver cells LO2 were cultured in Dulbecco’s modified Eagle’s medium (Gibco, Grand Island, NY, USA) supplemented with 10% fetal bovine serum (FBS) (Sijiqing Biotechnology Co., Hangzhou, China), 100 U/mL of penicillin and 100 μg/mL of streptomycin in a humidified atmosphere with 5% CO_2_ at 37 °C. Cells were cultured via weekly passage to ensure they were within the early ten generations and utilized for experimentation at 60%–80% confluency.

### 3.6. Flow Cytometry Experiments

Annexin-V-FITC/PI double staining assay was performed to detect apoptosis of LO2 cells as previously described [48,49]. The cells were seeded in 6-well plates at a density of 1 × 10^4^ cells/well and incubated for 24 h, and divided into control group, GS-1 and GS-2 groups. The normal control group was incubated with medium alone, and GS-1 and GS-2 groups were exposed to GS-1 and GS-2 at the concentration of 2.97 and 5.94 mg/mL, respectively. After treatment for 48 h, the cells both floating and attached were gently collected and centrifuged before being washed with cold PBS (Beijing Solarbio Science and Technology Co., Ltd., Beijing, China). The supernatant was removed and the cells were stained with 5 μL of Annxin-V-FITC and 10 μL of PI for 10 min at room temperature in the dark according to the manufacturer’s instructions (Nanjing KeyGen Biotech. Co., Ltd., Jiangsu, China). The cells were analyzed with a flow cytometer (FACSCalibur, BD Instruments Inc., Franklin Lakes, NJ, USA) and FlowJo 7.1.0 software. All experiments were performed in triplicate, and for each measurement, at least 20,000 cells were counted.

### 3.7. High Content Screening (HCS) Experiments

The toxic effects of GS-1 and GS-2 at different doses on LO2 cells were assayed by Assay Scan VTI HCS Reader (Thermo Fisher Scientific Corporation, Waltham, MA, USA) with the Cellomics^®^ Multiparameter Cytotoxicity 3 Kit (Thermo Fisher Scientific Inc.). The principle of the assay was that cells were labeled with flurorescent dyes that would indicate the cellular properties of interest, including nucleus, cell membrane permeability and mitochondrial membrane potential (*ΔΨ*m) and cytochrome c. All procedures were performed according to the manufacturer’s instructions. LO2 cells were seeded in 96-well plates (Costar, Corning Inc., Corning, NY, USA) at a density of 1 × 10^3^ cells/well and incubated for 24 h, and then exposed to sample solutions at the concentration of 1.48, 2.97 and 5.94 mg/mL, respectively. After treatment for 48 h, cells were washed twice with PBS and added membrane permeability and mitochondria membrane potential (*ΔΨ*m) dyes to each well, and then plates were sealed and ran immediately on the Assay Scan VTI HCS Reader to acquire images. Images were analyzed with HCS software. The nuclear size, cell membrane permeability intensity, mitochondrial membrane potential (*ΔΨ*m) intensity and the release of cytochrome c from mitochondria were calculated, respectively.

### 3.8. Western Blotting Experiments

Proteins were resolved on SDS-PAGE and detected as described previously [50,51]. The following antibodies were used in our studies: anti-(cleaved)-caspase-3, anti-(cleaved)-caspase-9, anti-cytc-m and anti-cytc-c were purchased from Santa Cruz Biotechnology, Inc. (Santa Cruz, CA, USA).

### 3.9. Elisa Experiments

The measurement of caspase-3 and caspase-9 activities was performed according to the manufacturer’s instructions. Briefly, treated cells were washed twice with ice-cold PBS and then were used for caspase-3 and caspase-9 activities assay (Nanjing Jiancheng Bioengineering Institute, Nanjing, China). The activity of caspase-3 and caspase-9 were quantified spectrophotometrically at 405 nm using ELISA reader (Nanjing, China).

### 3.10. Statistical Analysis

All experimental values were presented as means ± SD. The results were analyzed using one-way ANOVA followed by *LSD* tests, and Students’s *t*-test was used for the two groups’ comparison as needed. Values of *p* < 0.05 were considered to be statistically significant.

## 4. Conclusions

As a toxic Chinese medicinal herb, kansui has to be stir-baked with vinegar to reduce its toxicity for oral administration. Studies on LO2 cell have shown that the treatment with vinegar reduces the hepatotoxicity induced by kansui [15]. However, the potential mechanisms of this reduction require further study. Our previous research has shown that the EtOAc extract contains the main hepatotoxic fraction of kansui, and there are many toxic terpenoids including diterpenoids and triterpenoids in this EtOAc extract [20,52,53], so the EtOAc fraction of kansui was selected as our study object. The six terpenoids in the EtOAc extract of GS-1 and GS-2, 5-*O*-(2'*E*,4'*E*-decadienoyl)ingenol (**1**), 20-*O*-(2'*E*,4'*E*-decadienoyl)ingenol (**2**), 3-*O*-(2'*E*,4'*Z*-decadienoyl)-5-*O*-acetylingenol (**3**), 3-*O*-(2'*E*,4'*E*-decadienoyl)-20-*O*-acetylingenol (**4**), epi-kansenone (**5**), and 3-*O*-(2,3-dimethylbutanoyl)-13-*O*-dodecanoylingenol (**6**) were shown to contribute the most to the difference between GS-1 and GS-2. Taking into account their well-known toxicity [20], the decrease of these compounds’ contents in GS-2 might well explain how stir-baking with vinegar could reduce the toxicity of kansui. Of course, there are other compounds that also contribute to the difference between GS-1 and GS-2 shown in Figure 3, and coinciding with the literature [54], the contents of jatrophane-type diterpenoids were decreased after processing with vinegar. These results suggest that the toxic terpenoids, including jatrophane-type diterpenoids and 8-ene-7-one triterpenoids, and the decrease of their contents after processing with vinegar would be associated with the reduction in hepatotoxicity of kansui stir-baked with vinegar.

Mitochondria act as sentinels of diverse stress signals that emanate from other intracellular organelles, as well as a variety of environmental stress signals such as increased calcium levels, oxidative stress and low nutrient levels. In response to these signals, mitochondria become ruptured which leads to the release of pro-apoptotic molecules such as cytochrome c and induction of the cascade of apoptotic events such as formation of the apoptosome and activation of the initiator caspase-9 [55].

In this study results showed that the nucleus size and mitochondrial membrane potential *(ΔΨ*m) of LO2 cell were significantly decreased after treating by kansui, while the cytochrome c release, cell membrane permeability, activities of caspase-9 and caspase-3 were significantly increased. The results suggest that kansui could induce the LO2 cell apoptosis by stimulating the mitochondria, increasing the mitochondrial membrane permeability, and reducing the mitochondrial membrane potential leading to the release of proapoptotic proteins, such as cytochrome c, from the intermembrane space and consequently activating caspase-9 activity by combination cytochrome c with caspase-9 precursor molecule in the cytoplasm, furthermore, activating caspase-3 activity. Namely, kansui could induce hepatocyte apoptosis via mitochondrial pathway.

Compared with the kansui group, kansui stir-baked with vinegar could significantly increase the nucleus size and mitochondrial membrane potential (*ΔΨ*m) of LO2 cell, while decreasing cell membrane permeability, cytochrome c release, activities of caspase-9 and caspase-3. The results show that kansui stir-baked with vinegar could reduce the hepatotoxicity induced by kansui through effectively inhibiting LO2 cell apoptosis via mitochondrial pathway. That is, the reduction of apoptosis was achieved by decreasing the mitochondrial membrane permeability, raising the mitochondrial membrane potential (*ΔΨ*m) and inhibiting the release of cytochrome c, next, reducing the activation of caspase-9 by decreasing the combination cytochrome c with caspase-9 precursor molecule in the cytoplasm, and finally, by reducing the activity of caspase-3.

Therefore, this work has revealed the differentiating components and the intrinsic pathway of cell apoptosis in human normal liver cells LO2 to study the mechanism of the reduction in hepatotoxicity of kansui stir-baked with vinegar: decreased contents of toxic terpenoids and inhibition of the intrinsic pathway of cell apoptosis via blockade of mitochondrial cytochrome c release and caspase activation. Moreover, all of these results should be helpful to reveal the mechanism of kansui hepatotoxicity, and the mechanism of reduction in hepatotoxicity of the kansui stir-baking with vinegar, furthermore, to effectively guiding safer and better clinical application of this herb.
